# A Validated Methodological Approach to Prove the Safety of Clinical Electromagnetic Induction Systems in Magnetic Hyperthermia

**DOI:** 10.3390/cancers16030621

**Published:** 2024-01-31

**Authors:** Maria Anastasia Rouni, Boaz Shalev, George Tsanidis, Ioannis Markakis, Sarah Kraus, Pazit Rukenstein, Doron Suchi, Ofer Shalev, Theodoros Samaras

**Affiliations:** 1 Thessaloniki Software Solutions S.A., 55535 Thessaloniki, Greece; tsanidis@thess.com.gr (G.T.); jmarkakis@thess.com.gr (I.M.); 2 Faculty of Sciences, School of Physics, Aristotle University, 54124 Thessaloniki, Greece; theosama@auth.gr; 3 New Phase Ltd., Petah Tikva 4934829, Israel; boaz@newphase.co.il (B.S.); sarahk@newphase.co.il (S.K.); pazitr@newphase.co.il (P.R.); dorons@newphase.co.il (D.S.); ofers@newphase.co.il (O.S.); 4 Department of Physics, University of Malta, 595 38 Msida, Malta

**Keywords:** magnetic nanoparticle hyperthermia, anatomical human model, Sim4Life, Pennes BioHeat equation, temperature-dependent perfusion

## Abstract

**Simple Summary:**

This study examines the application of magnetic nanoparticle hyperthermia (MNH), a cancer treatment technique that utilizes magnetic particles at the scale of nanometers and a controlled magnetic field to selectively heat and destroy cancer cells. The study focuses on a specific system, the Sarah Nanotechnology System, which combines these magnetic particles and a device that generates the magnetic field. The main goal is to ensure this treatment is safe for patients. We used a combination of real-world experiments and computer simulations to test how the system affects the body’s temperature, particularly aiming to avoid overheating healthy tissues. We used a virtual human model to predict temperature changes during treatment. The findings are promising for safely using this advanced technology in cancer treatment, potentially offering a new, targeted approach for patients with advanced-stage tumors. This could be a significant step forward in cancer therapy, highlighting the importance of combining experimental and computational methods in medical research.

**Abstract:**

The present study focuses on the development of a methodology for evaluating the safety of MNH systems, through the numerical prediction of the induced temperature rise in superficial skin layers due to eddy currents heating under an alternating magnetic field (AMF). The methodology is supported and validated through experimental measurements of the AMF’s distribution, as well as temperature data from the torsos of six patients who participated in a clinical trial study. The simulations involved a computational model of the actual coil, a computational model of the cooling system used for the cooling of the patients during treatment, and a detailed human anatomical model from the Virtual Population family. The numerical predictions exhibit strong agreement with the experimental measurements, and the deviations are below the estimated combined uncertainties, confirming the accuracy of computational modeling. This study highlights the crucial role of simulations for translational medicine and paves the way for personalized treatment planning.

## 1. Introduction

Magnetic nanoparticle hyperthermia (MNH) is a minimally invasive therapeutic technique for targeted heating with applications in cancer treatment [1,2], utilizing the unique properties of magnetic nanoparticles (MNPs). Operating on the principle of converting magnetic energy into heat within an oscillating magnetic field, MNH allows for precise temperature control at the tumor site by adjusting the amplitude and frequency of these oscillations. Heat dissipation in tumor cells by MNPs mainly occurs through two mechanisms corresponding to the heat generation processes in magnetic materials. The first is related to hysteresis losses in bulk materials. The second mechanism corresponds to relaxation losses (Brown and Néel relaxations). The efficiency of these heat transfer processes depends on the specific design parameters and physical properties of the MNPs, such as size, composition, and magnetic characteristics.

During the implementation of MNH, a magnetic fluid, usually a dispersion of coated MNPs, is injected into the patient’s circulation or directly into the tumor [3]. When the MNPs are on site, the externally applied AMF generates a local temperature increase due to heating of the MNPs, resulting in the hyperthermic death of malignant cells.

However, the concurrent induction of eddy currents, resulting from a time-varying magnetic field in the body according to Faraday’s law of induction, and the consequent temperature increase in healthy tissues often cause local heating, leading to discomfort, pain, or distress in patients during treatment due to AMF exposure. Atkinson et al. [4] theoretically estimated the rate of heat production per unit of tissue volume for a cylindrical body and introduced the highest acceptable value of the product of the magnetic field strength H and frequency f of (H × f) at 4.85 × 10^8^ Am^−1^s^−1^. This value, known as the ‘Atkinson–Brezovich limit’ was supported by experiments performed with a coil operating at 13.56 MHz positioned around the thorax of patients. It was found that the patients could thermally tolerate magnetic fields up to 35.8 A/m. Since the heating power of the induced eddy currents is proportional to the square of the product (H × f × D), where D is the diameter of the eddy current loop, Hergt and Dutz [5] proposed a safety limit of 5 × 10^9^ Am^−1^s^−1^, which is one order of magnitude higher than that of Atkinson–Brezovich, for body parts of smaller diameters entering the treatment coil. It should be noted that the above calculations assume homogeneous tissue and do not take into account the effect of anatomical constrictions or tissue interfaces, which have been shown to result in local hotspots [6,7]. It appears from the literature that since the clinical study reported in [4], no other studies have been performed to assess the safety of coils intended for MNH use. A recent study examined safety for animals [8], for which much smaller diameters of coils are used than those for humans. Several studies have proposed different treatment strategies to mitigate healthy tissue heating by eddy currents. These strategies range from moving the coil [9,10,11] and intermittent magnetic field exposure [12] to the design of new coils [13,14]. Experiments with in vitro phantoms have shown that some of these strategies can considerably reduce the undesirable heating of healthy tissues.

An important issue that arises in the safety evaluation of MNH is the existence of a valid predictive model for temperature distribution, which can also be used in treatment planning and study design. Although there exist numerical studies on MNH modeling [14,15], only a few of them have been validated ex vivo [16] or in vivo [17]. To the knowledge of the authors, no validation study has been published so far for a predictive model of temperature rise in humans undergoing MNH in clinics.

The objective of the current study is to present, for the first time, a clinically validated computational model for temperature distribution inside the body of human patients exposed to MNH using a novel Electromagnetic Induction System (EIS) manufactured by New Phase Ltd. (Petah Tikva, Israel). The predictive model has been validated by clinical data obtained from a Phase I feasibility clinical study (MOH_2022-09-18_012060) [18], conducted in patients with stage IV solid tumors who signed an informed consent form and were treated with escalating doses of MNPs according to the NOAEL [19] criteria and AMF irradiation, to evaluate the safety of the system, which operates well above the Atkinson–Brezovich limit. Nevertheless, the model can also be used for treatment planning once a validated model for the tumor heating rate of the injected MNPs [19] is integrated into the computational calculations.

## 2. Materials and Methods

**Figure 1:**
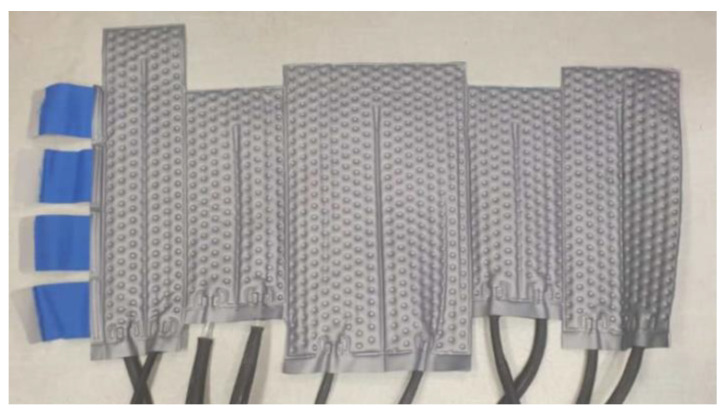
Cooling blanket system used for validation and for clinical trial.

The Sarah Nanotechnology System is a medical device developed by New Phase Ltd. to treat stage IV metastatic solid tumors through the delivery of thermal energy to malignant cells, thereby causing hyperthermic cancer cell death at sub-ablative temperatures [19,20]. The system involves two main components, MNPs named Sarah nanoparticles (SaNPs) and an Electromagnetic Induction System (EIS). The SaNPs, which contain an encapsulated superparamagnetic iron oxide core and paraffin wax as a phase change material that keeps the temperature of the nanoparticles at a maximum of 50 ± 3 °C, have an average size of 135 ± 10 nm and magnetic saturation above 60 emu/g. These MNPs are administered intravenously to the patient [21] and become localized via the Enhanced Permeability and Retention (EPR) effect [22] in cancerous tissues. Following the delivery and accumulation of the nanoparticles in the surrounding malignant tissue, the patient is placed in the center of the EIS coil and undergoes partial-body exposure with an AMF of 9 mT ± 1 mT at a frequency of 290 kHz ± 10%. The SaNPs convert electromagnetic energy to thermal energy, thereby heating the malignant cells they are in contact with and causing their hyperthermic cell death. To minimize unintended patient body surface heating, the system is accompanied by a cooling blanket system (CBS) (Figure 1), which includes a blanket filled with flowing water connected to a chiller and optical temperature probes to measure the skin temperature during AMF exposure. During treatment, the patient wears the CBS to cover the area of exposure, while the chiller keeps the water temperature constantly at 20 °C.

To numerically evaluate the thermal impact of the medical system on a representative patient, a triple validation process of the coil, CBS, and human model was followed, involving multiple experimental measurements and sequences of electromagnetic and thermal simulations. All the experiments, which included magnetic field and temperature measurements, were conducted on site by New Phase Ltd., implementing custom configurations and using the appropriate equipment (refer to Supplementary Material). The computational simulations were designed and run in the Sim4Life platform for electromagnetic simulations (Sim4Life 6.2, Zurich MedTech AG, Zurich, Switzerland) using the platform’s low-frequency (LF) and thermal solvers, which implement the Magneto Quasi-Static (M-QS) approximation and the Pennes BioHeat transfer equation (BHTE) [23], respectively.

### 2.1. Coil Simulations

**Figure 2:**
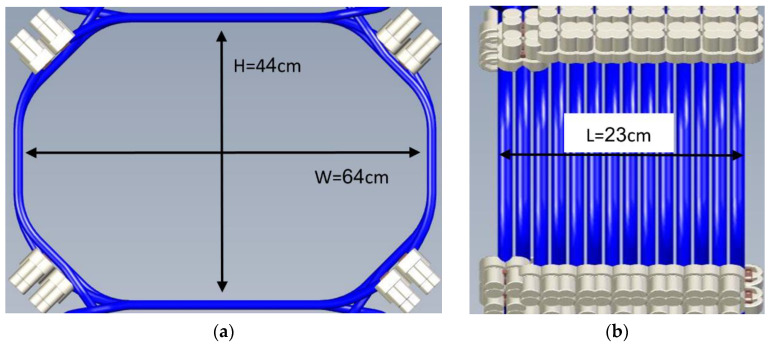
Coil CAD model (**a**) front view and (**b**) side view. The coil was modeled numerically in the Sim4Life platform as a current source, and it consisted of 14 turns. Each turn as circulated by a current of 316.19 A amplitude.

The EIS coil was initially modeled in the Sim4Life platform, following the CAD design provided by New Phase Ltd. (refer to Figure 2). The coil featured a total of 14 turns, each with an oval-like shape, and measured 44 cm in height, 64 cm in width, and 23 cm in length.

**Figure 3:**
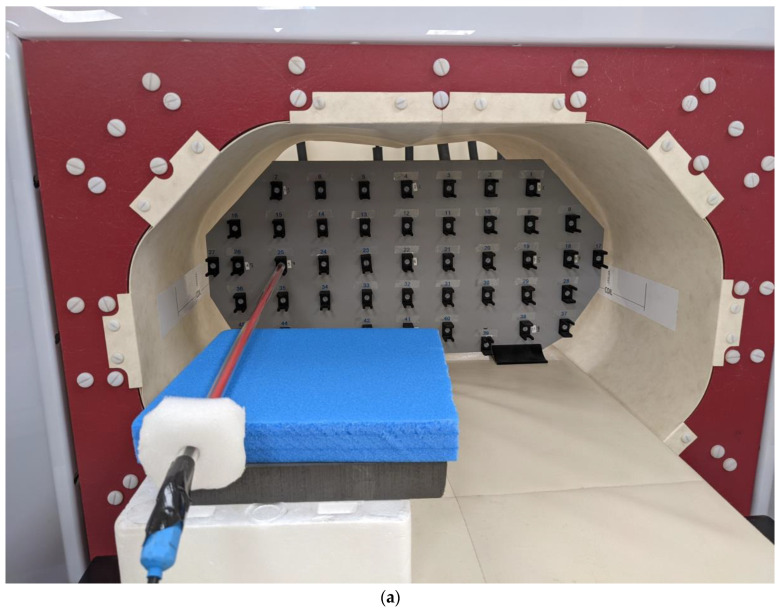

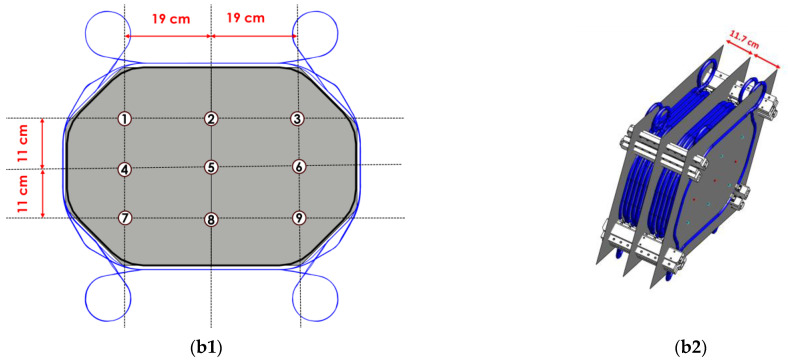
(**a**) Experimental configuration for the magnetic field measurements (**b1**). Illustration of measurement points in EIS coil (**b2**) over the selected planes inside the coil model.

In the M-QS simulation, the coil was represented as a current source operating at a frequency of 288 kHz and a current amplitude of 316.19 A, so that the numerically calculated magnetic field induced by the model would agree with the measured magnetic field value of 9 mT in the coil’s isocenter. The match between the actual EIS coil and the numerical model was confirmed through magnetic field measurements at various points on three axial planes, specifically, the isocenter, and planes 11.7 cm above and below it, as illustrated in Figure 3.

### 2.2. Agar Phantom Simulations

**Figure 4:**
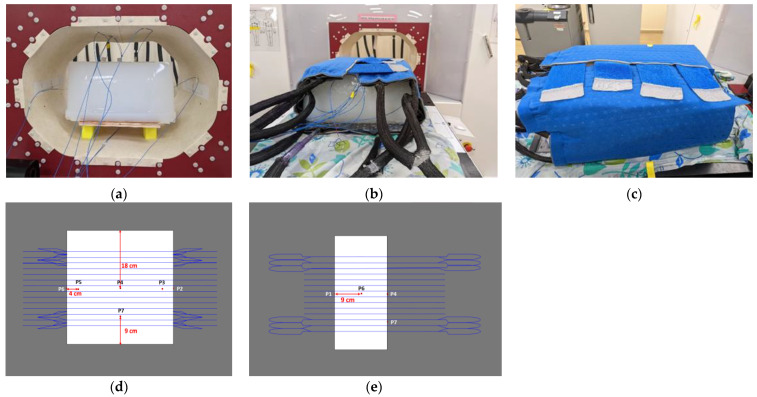
Measurement setup of the agar phantom inside the CBS: (**a**) front view without cooling blanket, (**b**) front view with cooling blanket, (**c**) side view and points of interest, (**d**) points where temperature probes were placed—coronal view, and (**e**) points where temperature probes were placed—side view.

Following the validation of the coil model, the next step was to measure the temperature impact of the CBS. For this purpose, an agar phantom (Supplementary Material) was employed to record temperature changes at seven locations using IR probes, as shown in Figure 4a–c.

Agar phantoms [13] were prepared using agar powder dissolved in a sodium chloride solution (27.36 g of NaCl in 40 L deionized water). The weight concentration of the agar in the phantoms was 1% (*w*/*w*). The phantoms had a square shape with a length of 36 cm, a height of 17 cm, and a width of 30 cm. The calculated electrical conductivity was 0.18 S/m and the measured was 0.2 S/m [16], similar to that of human skin tissue [24].

At the same time, a similar phantom was digitally replicated in the Sim4Life platform, as shown in Figure 4d,e. The heat transfer coefficient for each section of the CBS, necessary for defining the boundary conditions in thermal simulations, was calculated. This calculation, based on the corresponding hydraulic diameters and the Nusselt numbers [25], led to an average value of 210 W/m^2^/K for all CBS’s sections.

Two scenarios were experimentally and computationally investigated to evaluate the effect of the CBS model. The first considered the CBS OFF, whereas the second considered the CBS ON. The same AMF exposure scheme, named [7-5-7], was implemented in both cases and involved three sequential steps. The applied protocol started with a 7 min cycle of heating with AMF irradiation turned on, followed by a 5 min break with AMF turned off, and then, an additional cycle of 7 min of heating with AMF turned on. In the “CBS ON” scenario, the same AMF exposure scheme was used [7-5-7] with the CBS ON throughout the entire exposure scheme of 7 + 5 + 7 = 19 min in total.

### 2.3. Human Phantom Simulations

**Figure 5:**
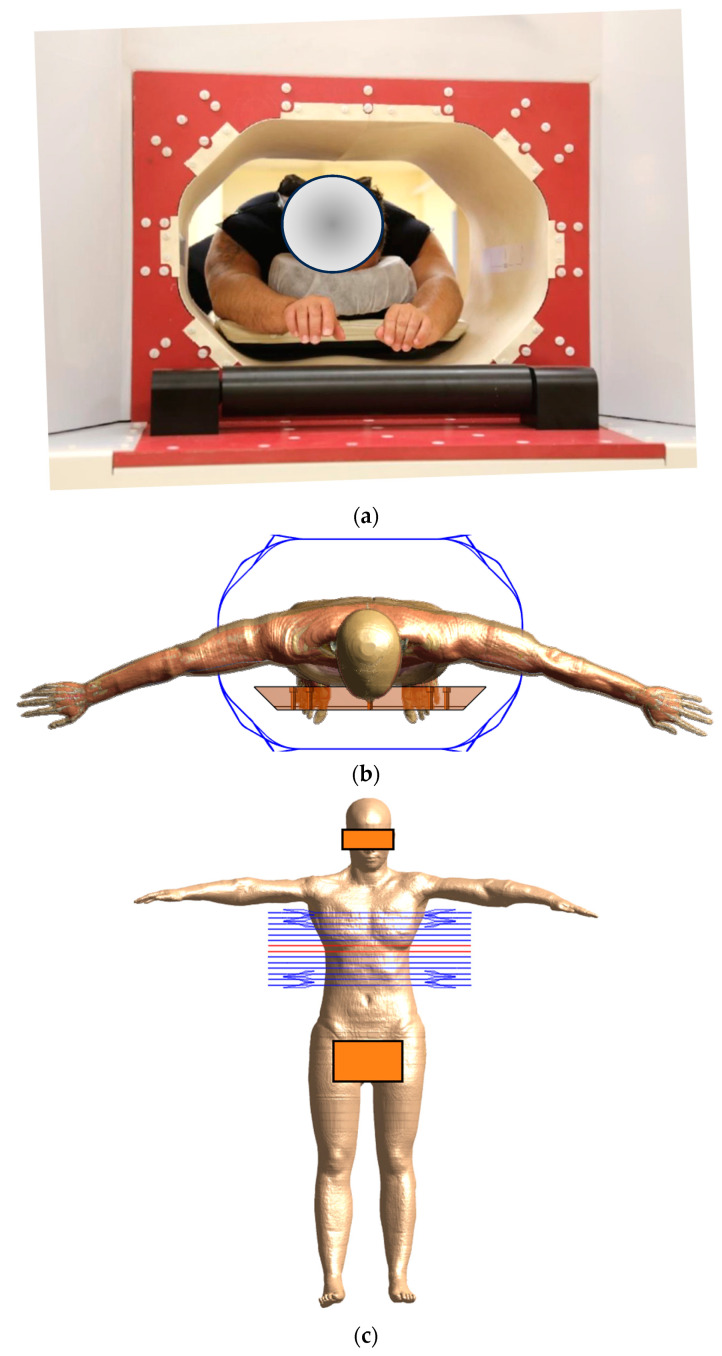
(**a**) Patient inserted in the Electromagnetic Induction System during the clinical trial; (**b**,**c**) human model of Ella placed inside the coil model with axial and trans axial views, respectively. The horizontal lines, in both blue and orange, depict the coil. More specifically, the orange loops delineate the coil’s isocenter plane.

The posable Ella model (Ella cV3-1, https://doi.org/10.13099/VIP11002-03-1 [26]) of the Virtual Population (ViP) family [6] was selected to serve as a typical treatment candidate for the MNH simulations, because it meets two major criteria. Firstly, the anthropometric characteristics of the model (Table 1), such as its Body Mass Index (BMI), are indicative of the target patient population, and secondly, its posing functionality allows us to mimic the actual clinical practice, where the patient’s arms remain outside of the coil area during AMF treatment. In the Sim4Life environment, Ella was consistently equipped with the CBS to replicate treatment conditions, and was placed in a prone position, with arms extended horizontally outside the coil, as shown in Figure 5. The dielectric parameters of the tissues in the Ella model were chosen according to the database of Gabriel [24].

In the simulation process, setting the appropriate perfusion parameters was a critical step. Rather than handling blood perfusion as a static parameter, a more realistic approach was adopted. This approach was based on the findings of Drizdal et al. [27], which highlighted the temperature-dependent behavior of blood perfusion during superficial hyperthermia. The results of Drizdal et al. suggest that blood perfusion exhibits dynamic variations with changes in temperature. To capture this variability, three distinct scaling factors (*SFs*) were integrated into the simulations. These factors, designed to adjust the baseline perfusion values at 37 °C for essential tissues such as skin, fat, and muscle, were applied in the BHTE as $SF\times \left[\rho_{b}c_{b}\omega \left(T-T_{b}\right)\right]$. The SFs for each tissue type were calculated as follows:

(1) $${SF}_{s(T)}=\left\{\begin{array}{ll} 1+9.2\exp\left(-\frac{{\left(T-44\right)}^{2}}{10}\right) & T\leq {44\;}^{{}^{\circ}}C\\ 10.2 & T>{44\;}^{{}^{\circ}}C \end{array}\right.$$

(2) $${SF}_{f(T)}=\left\{\begin{array}{ll} 1+\exp\left(-\frac{{\left(T-45\right)}^{2}}{12}\right) & T\leq {45\;}^{{}^{\circ}}C\\ 2 & T>{45\;}^{{}^{\circ}}C \end{array}\right.$$

(3) $${SF}_{m(T)}=\left\{\begin{array}{ll} 1+7.9\exp\left(-\frac{{\left(T-45\right)}^{2}}{12}\right) & T\leq {45\;}^{{}^{\circ}}C\\ 8.9 & T>{45\;}^{{}^{\circ}}C \end{array}\right.$$

The temperature data of six typical patients (detailed in Table 1), with all participants in a clinical trial approved by the Ethical Committee (Protocol: CL-100-001-R Rev 14, dated 23 October 2023, Helsinki RMS 0397-22, MOH: MOH_2022-09-18_012060, 202228263), were utilized in this study. Each participant provided their consent after signing an informed consent form. The inclusion requirements for the study were limited to patients with a maximum torso circumference of up to 110 cm, to ensure that they would fit within the system bore. This set of clinical data was used to validate Ella as a proxy (digital twin) for the patient population [7].

**Figure 6:**
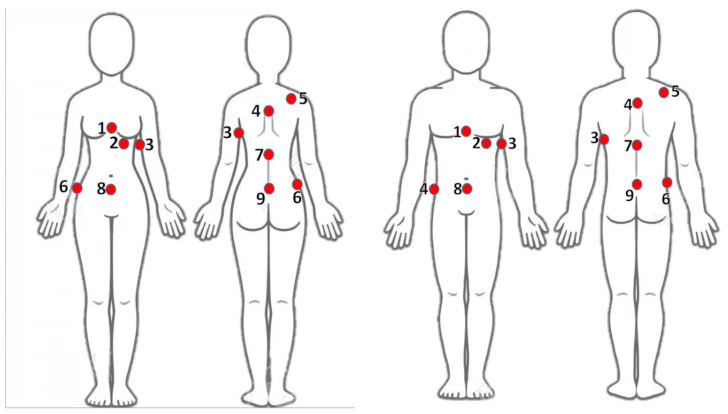
Temperature probe locations on female (**left**) and male (**right**) patients: Probe 1 (sternum), Probe 2 (inframammary fold), Probe 3 (right lung area), Probe 4 (upper back), Probe 5 (shoulder, reference), Probe 6 (waist), Probe 7 (mid-back), Probe 8 (abdomen), and Probe 9 (lower back).

The surface temperature of each patient was measured using optical temperature probes at nine strategic points (as illustrated in Figure 6) throughout the treatment with a sampling rate of 1 min, to ensure precise and consistent data collection. The selection of these probe locations was guided by the objective to obtain a wide picture of the temperature distribution in the torso area, particularly at anatomical landmarks where anatomical tissue narrowing occurs, as these points are expected to maximize hotspots due to eddy currents. Probe 1 was positioned on the sternum, and Probe 2 was located on the inframammary fold. Probe 3 was attached to the right lung area, while Probe 4 was placed on the upper back. Probe 5, used as a reference, was situated on the shoulder, outside the CBS. The waist was monitored by Probe 6, and Probe 7 was fixed to the mid-back. Probe 8 was placed on the abdomen, and finally, Probe 9 was positioned on the lower back. Additionally, the core temperatures of all patients were measured using an oral thermometer. The average readings varied from 35.5 to 37 °C during treatment, with the maximal allowable increase in temperature throughout the entire clinical session being 1.5 °C. The clinical session followed a [5-7-5] protocol according to the approved phase 1 clinical trial protocol, which stands for 5 min of heating, followed by 7 min of rest and 5 more minutes of heating.

The same treatment protocol, [5-7-5], was simulated with Ella with the CBS ON and the temperature was recorded at the locations of the experimental optical temperature probes. Then, the computational thermal model was validated for all nine probe locations and the whole treatment time (17 min).

## 3. Results

### 3.1. Coil Validation

**Figure 7:**
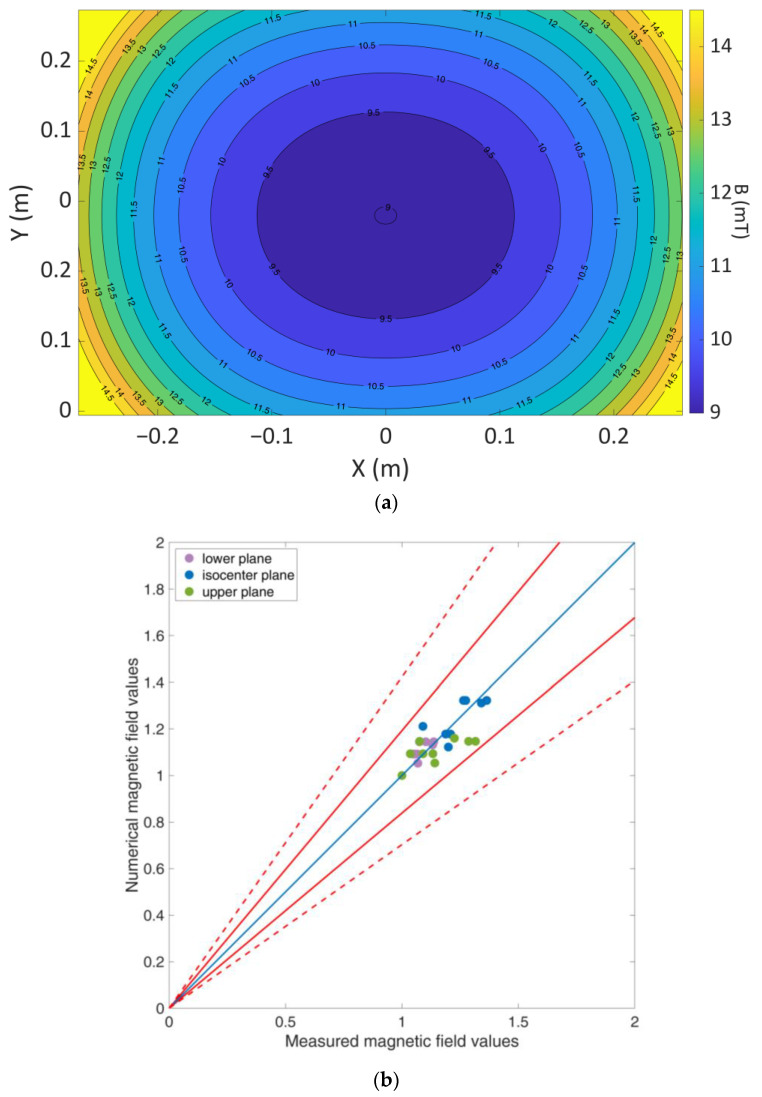
(**a**) Magnetic field distribution at the isocenter axial plane, based on numerical calculations. (**b**) Numerical magnetic field values normalized to planar central magnetic field value, plotted as a function of the normalized measured magnetic field values on three planes. The continuous lines correspond to the combined standard uncertainty, whereas the dashed lines signify the combined extended uncertainty. Blue line represents the identity 1:1 line.

The distribution of the induced magnetic field is shown in Figure 7a. Figure 7b presents a comparison between the numerical and measured values, normalized to the central value of each of the three planes involved in the validation process. When these values are plotted against each other, they fall within the acceptance limits, as determined by the estimated combined standard/expanded uncertainty, detailed in the Supplementary Materials [28,29].

### 3.2. Cooling System Validation

**Figure 8:**
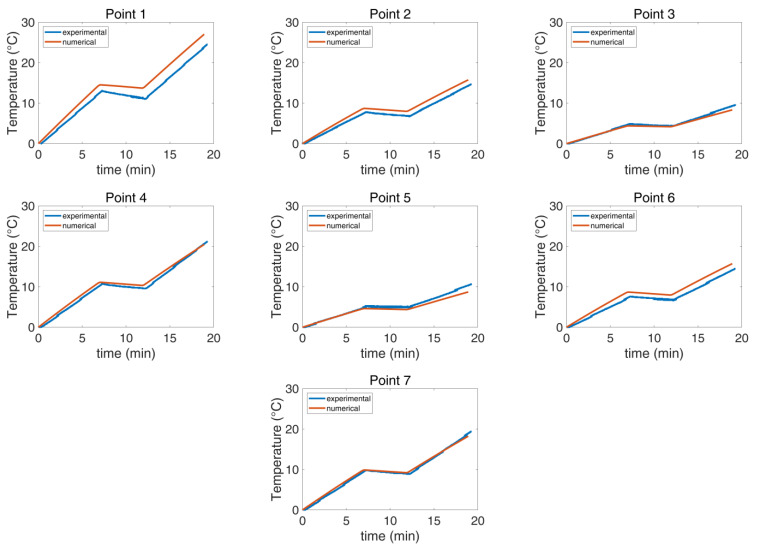
Comparison of the experimental and numerical temperature per selected point when the CBS is OFF The maximum deviation is 18%.

**Figure 9:**
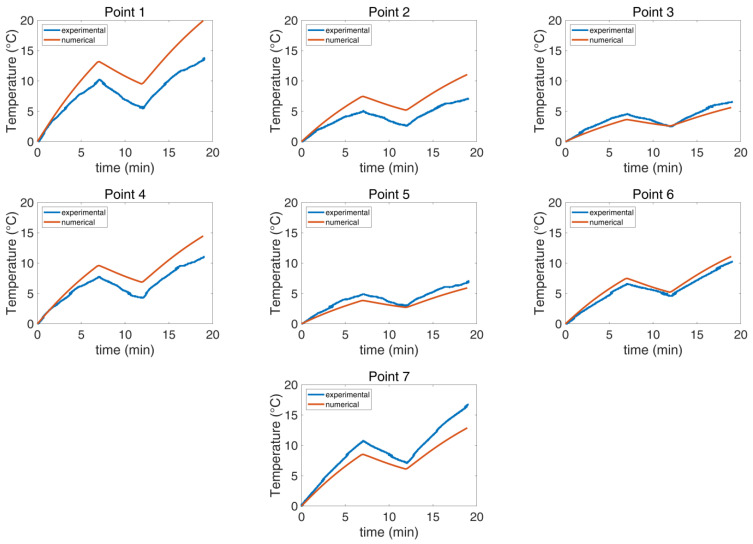
Comparison of the experimental and numerical temperature per selected point when the CBS is ON. The maximum deviation is 56%.

The experimental and the numerical agar phantom temperatures were compared as functions of time and temperature for the seven selected points (Figure 4). The measurements and the simulation results showed good agreement in both investigated scenarios, as detailed in Table 2, with the maximum deviation at the final temperature reached when the CBS was OFF being 18% (Figure 8), whereas the corresponding value with the CBS ON was 56% (Figure 9). The deviation for both cases was lower than the estimated combined standard/expanded uncertainty (see Supplementary Material). Notably, while in Figure 8, the numerical and experimental data align well, Figure 9 exhibits notable discrepancies at points 1, 2, and 4. These deviations are likely due to some random displacement of the thermal probes when the blanket was put on. This interpretation is further supported by the fact that point 6, which is symmetrical to point 2, does not exhibit similar deviations.

### 3.3. Human Model Validation

**Figure 10:**
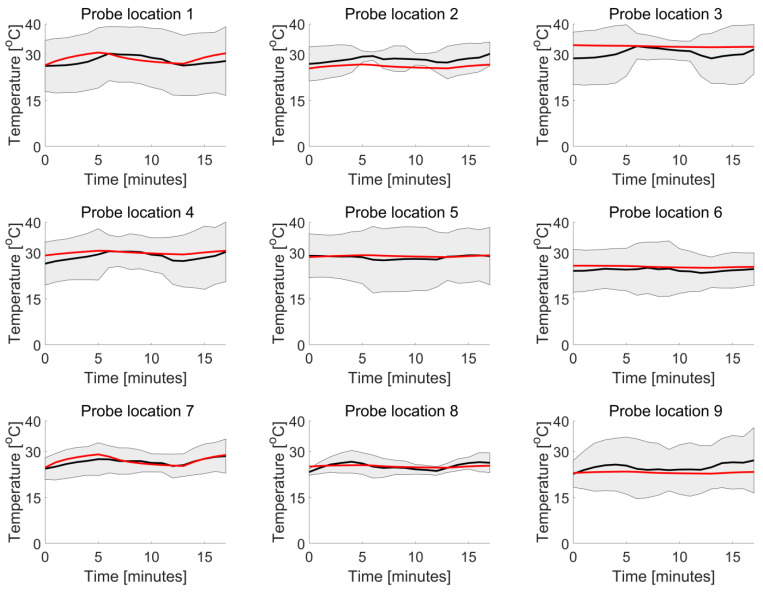
The black line represents the average measured temperature, typically within two standard deviations (grey area), compared to the numerically calculated temperature of Ella (red) during the treatment session and across the nine probe locations.

The results demonstrate that the numerically calculated temperature of Ella is quite similar to the average measured temperature of the six patients for every probe location during the entire session (Figure 10). Integral to this analysis, the SAR estimate for the Ella model, expressed as the peak spatial value averaged over 10 g (psSAR10g) of skin, was calculated numerically at 102 W/kg.

In Figure 10, the numerical and experimental results show good correspondence overall. However, a deviation is observed at probe location 2, situated in the chest area, specifically under the breasts. This discrepancy can be attributed to the unique anatomical features that influence the effectiveness of the cooling blanket. Unlike the simulation where the blanket is modeled to fit perfectly around Ella, the real-world scenario features a non-conformal blanket. This leads to reduced efficiency in heat transfer.

Furthermore, the error ${err}_{i}^{j}$ was calculated every minute for every probe location *i* and for every minute *j* as

(4) $${err}_{i}^{j}=T_{i}^{j,\;\;\;mean}-T_{i}^{j,\;\;\;Ella},$$

i.e., as the difference between the computationally and experimentally obtained temperature values, where for the experimental temperature value at each time point *j* and probe location *i*, the arithmetic mean, $T_{i}^{j,mean},$ over all six patients was used.

The computational relative standard uncertainty, $u_{i}^{j,\;num},$ was evaluated using a Type B [30] approach and sensitivity analysis at 10.97%. Therefore, for every numerically calculated temperature $T_{i}^{j,\;Ella}$, the standard uncertainty was evaluated as

(5) $$u_{i}^{j,\;\;\;num}=0.1097\times T_{i}^{j,\;\;\;Ella}$$

The experimental standard uncertainty $u_{i}^{j,exp}$ was evaluated using a Type A [30] approach as the standard error across all patients.

**Figure 11:**
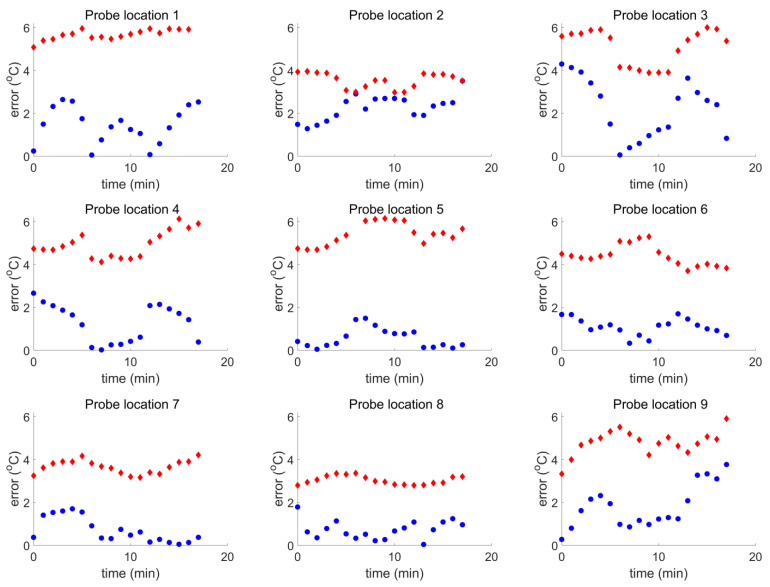
The blue data points represent ${err}_{i}^{j}$ and the red data points represent the quantity $\sqrt{{{(u}_{i}^{j,num})}^{2}+{\left(u_{i}^{j,exp}\right)}^{2}}$ of Equation (6).

Figure 11 shows that for all probe locations *i* and throughout the treatment (for all minutes *j*), the following condition was true:

(6) $${err}_{i}^{j}<\sqrt{{\left(u_{i}^{j,\;\;\;num}\right)}^{2}+{\left(u_{i}^{j,\;\;\;exp}\right)}^{2}}$$

The smallest validation margin (largest error) appears in Figure 11 for probe location 2 (front left of the chest) and the largest validation margin for probe location 5 (back left shoulder).

Therefore, it is shown that the thermal model of Ella is a validated model for the patient group which participated in the clinical study and had been chosen following specific selection criteria related to the anthropometric data of Table 1.

## 4. Discussion

Hyperthermia Treatment Planning (HTP) has increasingly made its way into clinical use in recent years. Current alternative treatments in MNP hyperthermia primarily focus on localized therapy, where tumors are identified, located, and then, directly injected with MNPs for targeted irradiation, as exemplified in systems, like the one by MagForce AG [31], that involve HTP [32]. The MNH system presented in this study introduces a method of regional irradiation, treating the patient’s entire torso, thus eliminating the need for precise tumor detection and localization. This approach, combined with the intravenous injection of MNPs, which accumulate around tumors due to the EPR effect [20], facilitates the treatment of multiple tumors, including undetected micro-tumors, across various torso locations, thus not being organ specific.

The strength of numerical simulations lies in their ability to evaluate the safety and effectiveness of different scenarios before actual treatment, allowing for patient selection, specific power excitation planning, and clinical outcome prediction [33]. These simulations are also crucial for training, treatment visualization, and basic research to enhance our understanding.

To our knowledge, this is the first introduction of a comprehensive methodology designed for assessing the safety of MNH treatment in the torso and founded on the validation of all key components (the treatment coil, the cooling system, and a detailed human anatomical model) with experimental data. Previous computational studies in the field of hyperthermia treatment often limited their scope to specific areas, such as the head and neck [34], focused on specific organs or animals [16], or employed simplified anatomical models based on the segmentation of a limited number of tissues [35], focusing on different hyperthermia modalities and on other (usually higher) operating frequencies. Our study, incorporating Ella as a digital proxy that closely mirrors the anthropometric characteristics of the target patient population, emphasizes the practical relevance and applicability of this approach.

The validation of the 3D models of human thermoregulation requires temperature data from various locations. The lack of temperature data from inside the body, such as muscle and fat temperatures, often makes validation challenging and leads to a limited understanding of fundamental thermoregulatory mechanisms [36]. However, it can be shown from the data in the current study, as well as from other similar validation studies in MRI safety [7,37], that the change in blood perfusion with increasing temperature must always be considered in the patient’s thermal model to achieve an accurate representation of the clinical situation. In a previous study, Murbach et al. [7] used two high-resistance temperature probes placed at numerically estimated temperature hotspot regions on both shoulders of a male subject inside a 64 MHz body coil. The authors showed that constant blood perfusion could overestimate the hotspot temperature by more than 3 °C. Oh et al. [37] used MRI thermometry to validate their thermal modeling of surface coils both in vitro (with an agar phantom) and in vivo (in a human forearm) in a methodological approach like ours. The regulation (increase) of local blood perfusion with temperature gave us a conservative value for the in vivo maximum temperature increase of about 25% greater than the value measured experimentally. However, the data on the temperature dependence of tissue thermal properties, including blood perfusion, are still scarce [38]. More experimental studies are necessary to collect detailed data for all tissues involved in the thermal modeling of MNH treatment.

As MNH methodologies evolve, predictive models like the one presented here will become indispensable. The future of thermal modeling looks promising, and as it matures, it will play an integral role in the medical field. With the advent of non-invasive thermometry approaches, thermal models will gain even more significance, especially where MR thermal imaging is not feasible. The validation of developed HTP tools in vivo and their integration into routine clinical workflows will be vital going forward [33].

Regarding the MNH treatment, the critical challenge has always been ensuring the targeted heating of malignant cells while safeguarding surrounding healthy tissues. Therefore, the discomfort arising from local heating due to AMF exposure accentuates the need for predictive models [36]. Systems like the one from New Phase Ltd., which operate beyond the established Atkinson–Brezovich limit [2], carry risks. Thus, our computational model, backed by clinical data from patients with stage IV solid tumors, offers an in-depth understanding of temperature distribution during MNH treatment and enables the expansion of irradiation treatment duration and/or power (Hxf).

## 5. Conclusions

This study introduces, for the first time, a clinically validated computational model that predicts temperature distributions in patients undergoing MNH treatments. The individual components of the model were validated in vitro, whereas the full thermal model of the patient was validated in clinical practice, considering both numerical and experimental uncertainties. This validation shows that the integration of temperature-dependent blood perfusion offers a more accurate physiological representation during treatment.

The clinical results and the predictions obtained by the validated computational model support the safe application of the New Phase MNH treatment. The temperature in healthy tissues of the torso does not reach harmful values, with the system preventing any thermal toxicity to patients. The inclusion criteria, which are dominated by the torso circumference of patients, may be broadened by using constant temperature monitoring.

Computational simulations play a pivotal role in translational medicine, not just in predictive modeling but also in calculating and validating safety and efficacy parameters. These simulations are instrumental in advancing clinical applications for MNH. While the primary focus of this research is clinical validation, it is obvious that the model’s potential extends to treatment planning.

## Figures and Tables

**Table 1:** Anthropometric characteristics of six patients and Ella model.

	Age [Years]	Weight [kg]	Height [cm]	BMI [kg/m^2^]	Back Width [cm]	Chest Height [cm]	Circumference [cm]	Gender
P01	55	52.0	160	20.3	26.0	17.0	82.0	F
P02	63	53.7	163	20.2	30.5	22.0	86.0	F
P03	64	61.0	160	23.8	38.0	17.0	86.5	F
P04	70	64.4	165	23.7	35.0	20.0	92.0	F
P05	56	47.2	155	19.6	N/A	N/A	93.0	F
P06	67	86.6	193	23.2	35.0	21.0	105.0	M
Ella	26	57.3	163	21.6	27.0	17.7	89.9	F

**Table 2:** Deviation in final temperature reached at each point between measurements and numerical simulations when the CBS is OFF and when the CBS is ON in terms of % relative error.

	Deviation						
	Point 1	Point 2	Point 3	Point 4	Point 5	Point 6	Point 7
CBS OFF	−10%	−7%	14%	4%	18%	−9%	6%
CBS ON	−46%	−56%	15%	−31%	13%	−8%	23%

## Data Availability

The data presented in this study are available in this article and supplementary material.

## Supplementary Materials

The following supporting information can be downloaded at: https://www.mdpi.com/article/10.3390/cancers16030621/s1, Table S1: Uncertainty budget of the magnetic field measurement system; Table S2: Uncertainty budget of the coil and phantom; Table S3: Uncertainty budget of the thermal probes measurement system; Table S4: Experimental uncertainty budget; Table S5: Uncertainty budget of numerical magneto-quasi-static simulations; Table S6: Uncertainty budget of numerical thermal simulations; Table S7: Uncertainty budget of numerical model; Table S8: Combined uncertainty of experimental measurements and numerical model.
