# Supplementary material for: A Validated Methodological Approach to Prove the Safety of Clinical Electromagnetic Induction Systems in Magnetic Hyperthermia
Source: Cancers (Basel). 2024 Jan 31;16(3):621. doi: 10.3390/cancers16030621 (PMC10854696; doi:10.3390/cancers16030621)
Supplement: Supplementary file 1 [file cancers-16-00621-s001.zip › cancers-2810322-supplementary.pdf]

Section S1. MATERIALS AND EQUIPMENT

This appendix provides a comprehensive list of the materials and equipment utilized in the current study. It includes both commercially available chemicals and specialized equipment, vital for the accurate execution and reproducibility of the research.

A. Chemicals and Phantoms

Agar powder for the preparation of agarose phantoms, catalog number 50004, was sourced from Lonza SeaKem® LE (Haifa, Israel), while NaCl was obtained from Sigma-Aldrich (Rehovot, Israel). AQUARIUS 101 water-based ultrasound gel was purchased from Dakar Medical (Nir Tzvi, Israel), and was used for gap filling and good contact between the patient’s torso to the cooling blanket.

B. Equipment for MNH Treatment and experimental measurements

The EIS was specially manufactured by UltraFlex Co. Ltd. (Sofia, Bulgaria) for New Phase Ltd. The CBS, comprising a wrap-around blanket and a chiller unit, was designed by New Phase Ltd. to conform to the human torso, and cool the patient’s skin during MNH treatment. Temperature monitoring was conducted using fiber optic temperature infrared (IR) probes from Optocon AG (Dresden, Germany), which operate at 2 Hz with a ±0.1ºC resolution. Additionally, a magnetic field probe was employed to accurately measure the field power distribution and frequency at specific points during the treatment process.

Section S2. UNCERTAINTY ASSESSMENT

Contained within this appendix is a technical analysis of uncertainties, focused on SAR evaluation and temperature simulations, deemed essential for the assurance of precision and reliability in the findings of the current study.

A. Grid Resolution Uncertainty for SAR Evaluation

The SAR was simulated with grid steps between 1 mm and 3 mm in Ella and phantom. The differences in maximum SAR values were less than 0.2dB.

B. Simulation Parameters for SAR Evaluation

The estimated deviation of the maximum SAR value considering variation of the dielectric properties of the tissues (±10%) is included in Supp. Table 5. Furthermore, comparison of simulations was made to investigate the impact of the exclusion of the legs and the head, simulating the whole body or only the torso, as was the case in this study. The 0.84 dB estimation is considered to be conservative as it corresponds to field variations in the proximity of the truncation region, and the domain of interest for this application is a more distant region; thus, the impact is expected to be lower.

C. Simulation Parameters for Temperature Evaluation

The temperature increase was simulated with grid steps between 1 mm and 2 mm. The differences were less than 0.02 dB. The thermal parameters of the tissues were varied assuming a variation of the thermal properties of the tissues by 10%. Their impact is approximately 0.15 dB.

Table S1 Uncertainty budget of the magnetic field measurement system

	Tolerance (dB)	Probability Distribution	Divisor	Std Uncertainty	Note
Probe calibration	0.58	N	1	0.58	measured
Probe position	1.64	R	$\sqrt{3}$	0.95	measured
Total std uncertainty	-	-	-	1.11	

Table S2 Uncertainty budget of the coil and phantom

	Tolerance (dB)	Probability Distribution	Divisor	Std Uncertainty	Note
Coil magnetic field	0.92	N	1	0.92	manufacturer's specifications
Coil frequency	0.35	N	1	0.35	manufacturer's specifications
Phantom (position)	0.43	R	$\sqrt{3}$	0.25	measured
σ	0.2	R	$\sqrt{3}$	0.12	(Neufeld *et al.*, 2009)
Total std uncertainty	-	-	-	1.07	

Table S3 Uncertainty budget of the thermal probes measurement system

	Tolerance (dB)	Probability Distribution	Divisor	Std Uncertainty	Note
Thermal sensor position	1.12	N	1	1.12	measured
Total std uncertainty	-	-	-	1.12	

Table S4 Experimental uncertainty budget

	Tolerance (dB)	Probability Distribution	Divisor	Std Uncertainty	Note
Magnetic field measurement system	1.11	N	1	1.11	Supp. Table S1
Coil & Phantom	1.07	N	1	1.07	Supp. Table S2
Thermal sensor position	1.12	N	1	1.12	Supp. Table S3
Total std uncertainty	-	-	-	1.91	

Table S5 Uncertainty budget of numerical magneto-quasi-static simulations

	Tolerance (dB)	Probability Distribution	Divisor	Std Uncertainty	Note
Computational space	1.45	R	$\sqrt{3}$	0.84	B
Grid resolution	0.17	N	1	0.17	A
Model positioning	0.10	N	1	0.10	A
Tissue parameters	0.29	R	$\sqrt{3}$	0.17	B
Total std uncertainty				0.88	

Table S6 Uncertainty budget of numerical thermal simulations

	Tolerance (dB)	Probability Distribution	Divisor	Std Uncertainty	Note
Grid resolution	0.01	N	1	0.01	C
Boundary conditions	0.04	N	1	0.04	C
Tissue parameters	0.15	N	1	0.15	C
Solver's uncertainty	0.3	N	1	0.3	(Cabot *et al.*, 2013)
Total std uncertainty				0.34	

Table S7 Uncertainty budget of numerical model

	Tolerance (dB)	Probability Distribution	Divisor	Std Uncertainty	Note
Numerical magnetic model	0.88	N	1	0.88	Supp. Table S5
Numerical Thermal model	0.34	N	1	0.34	Supp. Table S6
Total std uncertainty				0.94	

Table S8 Combined uncertainty of experimental measurements and numerical model.

	Tolerance (dB)	Probability Distribution	Divisor	Std Uncertainty	Note
Combined Experimental	---	---	---	1.91	Supp. Table S4
Combined Numerical	---	---	---	0.94	Supp. Table S7
Total (k=1)	---	---	---	2.13	
(k=2)	---	---	---	4.26	

References

Cabot, E. *et al.* (2013) 'Evaluation of the RF heating of a generic deep brain stimulator exposed in 1.5 T magnetic resonance scanners', *Bioelectromagnetics*, 34(2), pp. 104–113. Available at: https://doi.org/10.1002/bem.21745.

Neufeld, E. *et al.* (2009) 'Measurement, simulation and uncertainty assessment of implant heating during MRI', *Physics in Medicine and Biology*, 54(13), pp. 4151–4169. Available at: https://doi.org/10.1088/0031-9155/54/13/012.
